# Methodological developments in administrative data linkage for cross cutting policy relevant research: Working towards a sustainable data pipeline

**DOI:** 10.23889/ijpds.v8i2.2307

**Published:** 2023-09-14

**Authors:** Emma Turner, Jen Hampton, Rachel Huck, Clare Melson, Jason Powell-Bavester, Alissa Goodman, George Ploubidis, Robin Flaig, Andy Boyd

**Affiliations:** 1 University of Bristol, Bristol, United Kingdom; 2 ONS, London, United Kingdom; 3 University College London, London, United Kingdom; 4 University of Edinburgh, Edinburgh, United Kingdom

## Objectives

Develop administrative linkages within a national Trusted Research Environment (TRE) that hosts Longitudinal Population Study (LPS) data for over 20 LPS. We will describe the methodological development carried out to enable linkage to administrative datasets. These linked administrative data will support research for public good, informing policy and practice.

## Methods

The first sets of administrative data under consideration in this Feasibility Study are from the Department of Work and Pensions (DWP), the Department for Education (DfE) and HM Revenue and Customs (HMRC). Working with UK Government departments through a Task & Finish group we have gathered input from DWP, HMRC and DfE and Office for National Statistics (ONS) data sharing experts. The Task & Finish group identified three pragmatic data linkage and data sharing models, that would enable data to be linked via a newly designed secure data pipeline in a legal, secure, and trustworthy manner for all stakeholders.

## Results

To encourage sustainability and acceptability, a model designed to be maintained over a long period is based on the re-use of Departmental Personal Identifiable Information (PII) – i.e., name, date of birth, gender, National Insurance number - and attribute data already deposited by the Departments into ONS. ONS will develop for the linkage and extraction of ONS Data into the TRE a system which conducts, and quality assesses the linkage; minimises the Departmental data to participants within the TRE only and the variables specified in the agreements; and, de-identifies the data to their DEA processing standards. The minimised and functionally anonymous data extract will be securely transferred for ingest and integration into the TRE enabling researchers to address a wider range of questions for public benefit.

## Conclusion

This is a model for efficient and low-burden linkages to inform cross cutting research. It will form part of a responsive UK data science capability which can inform government research needs and be used to meet future crisis e.g. new pandemics, the impacts of climate change or economic shocks.

